# Low-Tidal-Volume Ventilation and Mortality in Patients With Acute Brain Injury: A Secondary Analysis of an International Observational Study

**DOI:** 10.1016/j.chest.2025.06.042

**Published:** 2025-07-08

**Authors:** Julian F. Daza, Doulia M. Hamad, Martin Urner, Kuan Liu, Sarah Wahlster, Chiara Robba, Robert D. Stevens, Victoria A. McCredie, Raphael Cinotti, Shaurya Taran, PROtective VENTilation Network, European Society of Intensive Care Medicine, Colegio Mexicano de Medicina Critica, AtlanRéa Group, Société Française d’Anesthésie-Réanimation–SFAR Research Network

**Affiliations:** a Division of General Surgery, Department of Surgery, University of Toronto, Toronto, ON, Canada; b Institute of Health Policy, Management and Evaluation, University of Toronto, Toronto, ON, Canada; c Interdepartmental Division of Critical Care Medicine, University of Toronto, Toronto, ON, Canada; d Department of Anesthesiology and Pain Medicine, University of Toronto, Toronto, ON, Canada; e Division of Biostatistics, Dalla Lana School of Public Health, University of Toronto, Toronto, ON, Canada; f Department of Neurology, University of Washington School of Medicine, Seattle, WA; g Department of Neurosurgery, University of Washington School of Medicine, Seattle, WA; h Department of Anesthesiology and Pain Medicine, University of Washington School of Medicine, Seattle, WA; i IRCCS Policlinico San Martino, Genoa, Italy; j Department of Surgical Sciences and Integrated Diagnostics, University of Genoa, Genoa, Italy; k Department of Anesthesiology and Critical Care Medicine, Johns Hopkins University School of Medicine, Baltimore, MD; l Department of Neurosurgery, Johns Hopkins University School of Medicine, Baltimore, MD; m Department of Radiology, Johns Hopkins University School of Medicine, Baltimore, MD; n Department of Biomedical Engineering, Whiting School of Engineering, Johns Hopkins University, Baltimore, MD; o UMR 1246 (Methods in Patient-Centered Outcomes and Health Research; SPHERE), Nantes Université (Nantes) and Tours Université (Tours), Nantes, France; p Department of Anesthesia and Critical Care, CHU Nantes, Nantes Université, Hôtel-Dieu, Nantes, France

**Keywords:** acute brain injury, low-tidal-volume ventilation, lung-protective ventilation, neurocritical care

## Abstract

**Background:**

Low-tidal-volume ventilation (LTVV) improves outcomes in critically ill patients, but its impact in patients with acute brain injuries (ABIs) is less certain.

**Research Question:**

What is the association between LTVV and mortality in mechanically ventilated patients with ABI?

**Study Design and Methods:**

We did a secondary analysis of a prospective observational study (NCT03400904; https://clinicaltrials.gov/study/NCT03400904). We compared LTVV (≤ 8 mL/kg predicted body weight [PBW]) with tidal volumes > 8 mL/kg PBW over the first 7 days of mechanical ventilation. Alternate analyses used lower thresholds for LTVV. Marginal structural Cox models were used to evaluate the association between LTVV and ICU mortality up to 60 days. Stabilized inverse probability treatment and censoring weights were generated using multivariable logistic regression adjusted for baseline and time-dependent confounders.

**Results:**

A total of 1,510 patients from 73 ICUs across 18 countries were included. The mean age was 52 years, 513 patients (34.0%) were female, and the most common ABI etiology was traumatic brain injury (n = 726; 48.1%). ARDS developed in 137 patients (9.2%). In patients receiving LTVV, adjusted incidence of ICU mortality was 40.2% (95% CI, 19.2%-61.1%), vs 59.7% (95% CI, 44.0%-75.4%) in patients receiving tidal volumes > 8 mL/kg PBW (marginal hazard ratio, 0.54; 95% CI, 0.33-0.88). There was no heterogeneity of treatment effect in subgroup analyses, and sensitivity analyses for unmeasured confounding yielded similar results. However, associations were less clear at lower thresholds of LTVV.

**Interpretation:**

In this predominantly non-ARDS cohort of patients with ABI, LTVV over the first 7 days of mechanical ventilation was associated with lower ICU mortality up to 60 days, vs tidal volumes > 8 mL/kg PBW. Future research should investigate effects in patients with ABI and ARDS, use of lower LTVV thresholds, and impact on additional end points including functional outcomes and adverse events.

## Graphical Abstract

**Figure:**
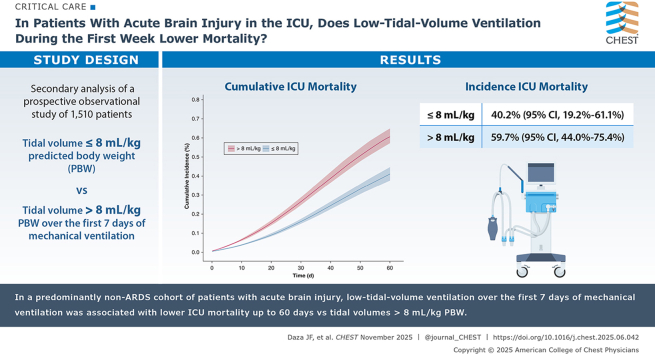

FOR EDITORIAL COMMENT, SEE PAGE 1079

**Take-Home Points**

**Study Question:** In patients with acute brain injury admitted to the ICU, what is the association between low-tidal-volume ventilation (≤ 8 mL/kg predicted body weight) during the first week of mechanical ventilation and ICU mortality?

**Results:** Patients ventilated with low tidal volumes had a lower adjusted risk of ICU mortality compared with those ventilated with > 8 mL/kg predicted body weight (marginal hazard ratio, 0.54; 95% CI, 0.33-0.88).

**Interpretation:** In this cohort of patients with acute brain injury, low-tidal-volume ventilation over the first 7 days of mechanical ventilation was associated with reduced ICU mortality, meriting further investigation in a clinical trial.

Low-tidal-volume ventilation (LTVV; defined as a tidal volume [Vt] of 4-8 mL/kg predicted body weight [PBW]) is a cornerstone of care in patients with ARDS.^1^^,^^2^ When combined with other lung-protective strategies, LTVV reduces morbidity and mortality by minimizing ventilator-induced lung injury (VILI).^3^, ^4^, ^5^ For patients without ARDS, LTVV increases the proportion of transplanted lungs available from brain-dead donors,^6^ reduces pulmonary and nonpulmonary complications after intraabdominal surgery,^7^ and limits the downstream occurrence of ARDS in patients receiving invasive ventilation for diverse conditions.^8^

Patients with acute brain injury (ABI) may also benefit from LTVV,^9^ but uptake in this population may be limited by concerns about intracranial hypertension and inability to perform a neurologic examination, due to the need for additional sedation or neuromuscular blockade (often required to facilitate synchronous ventilation).^10^ Most landmark ARDS trials excluded patients with ABI,^3^^,^^11^^,^^12^ and existing guidance for Vt selection in the ABI population is based on indirect evidence.^10^ A systematic review of 8 observational studies including 5,639 patients with ABI found no significant association between LTVV (Vt < 8 mL/kg PBW) and short-term mortality. Although a 2024 multicenter randomized trial in patients with ABI found worse short- and long-term outcomes with LTVV (Vt = 6 mL/kg PBW) compared with Vt ≥ 8 mL/kg PBW,^13^ its clinical implications remain debated.^14^ Therefore, the optimal Vt in this population remains uncertain.

In the present analysis, we used observational data and causal inference methods that account for time-varying confounding to examine whether LTVV is associated with ICU mortality in patients with ABI. Standard regression methods present important shortcomings in this context, because they cannot sufficiently handle time-varying confounders that predict subsequent levels of the treatment or act as mediators for the outcome.^15^^,^^16^ Prior observational studies in ABI populations have evaluated LTVV as a static exposure,^9^^,^^17^ but this does not fully capture the dynamic nature of ventilator management in clinical practice.^18^ Therefore, we used marginal structure models to evaluate the association between LTVV (delivered as a dynamic treatment over the first 7 days of mechanical ventilation) and mortality in patients with ABI. This secondary analysis was conducted using data from a prospective international study in which most patients did not have ARDS at the onset of mechanical ventilation.^19^

## Study Design and Methods

This was a secondary analysis of the Extubation in Neurocritically Ill Patients and Association with Outcomes (ENIO) prospective observational study.^19^ Ethics approval was granted by the research ethics boards of Nantes University (REB 23-153-12-180). Reporting followed the Strengthening the Reporting of Observational Studies in Epidemiology statement (e-Appendix 1 in the online article).^20^

### Study Cohort

Details of the original study cohort are published in full elsewhere.^19^ Briefly, consecutive patients aged ≥ 18 years with ABI (traumatic brain injury, subarachnoid aneurysmal hemorrhage, intracranial hemorrhage, ischemic stroke, brain tumor, central nervous system infection) were recruited from 73 ICUs across 18 countries between June 2018 and November 2020. All patients received invasive ventilation for at least 24 hours. Data were collected on patient demographics, comorbidities, ABI diagnosis, treatments, and clinical outcomes. Mechanical ventilation and gas exchange variables were recorded on days 1, 3, and 7 of invasive ventilation. Available data included mode of ventilation (spontaneous or assist-controlled), Vt (mL), plateau pressure (P_plat_; cm H_2_O), positive end-expiratory pressure (PEEP; cm H_2_O), respiratory rate (breaths/min), Pao_2_ (mm Hg), Paco_2_ (mm Hg), and pH.

### Exposure

The exposure for the present analysis was LTVV, defined as a Vt ≤ 8 mL/kg PBW and calculated using the patient’s sex and height as per the ARDS Network equation.^3^ We chose a threshold of 8 mL/kg PBW for the primary analysis, as this is the value commonly used to define lung-protective ventilation in past and present ARDS guidelines.^1^^,^^2^^,^^21^ The comparator was Vt > 8 mL/kg PBW. The exposure was measured on days 1, 3, and 7 from the initiation of mechanical ventilation. To account for shifts over time in clinician comfort with using lower Vt in patients with ABI,^22^^,^^23^ we repeated the analysis using longitudinal exposure profiles for Vt with thresholds of 7.5, 7, 6.5, and 6 mL/kg PBW. We chose not to evaluate exposure thresholds below 6 mL/kg PBW due to potential concerns related to further reducing Vt in patients with ABI (eg, resulting from effects of hypercarbia on intracranial pressure [ICP]).^24^

### Primary End Point

The primary end point was ICU mortality, which included deaths from withdrawal of life-sustaining therapies. Our calculation of this end point assumed that all deaths occurred in close temporal proximity with the decision to withdraw.^25^^,^^26^ Patients were followed from initiation of mechanical ventilation (ie, time zero) until death or ICU day 60, whichever occurred earliest. Censoring occurred at ICU discharge. Patients who developed ARDS were also censored because they would be unlikely to continue receiving Vt > 8 mL/kg PBW. As the timing of ARDS onset was not recorded in the ENIO study, we assumed that ARDS occurred within the first 3 days of invasive mechanical ventilation; this approximation is consistent with prior data in the ABI population.^9^^,^^27^^,^^28^

### Adjustment Covariates

Confounders for the association between LTVV and mortality were selected on the basis of clinical relevance and subject matter knowledge. Time-fixed confounders included demographic characteristics, medical comorbidities, etiology of ABI, and severity of brain injury as estimated by the lowest Glasgow Coma Scale (GCS) score before endotracheal intubation. Time-varying confounders included ventilator mode, respiratory rate, P_plat_, Pao_2_/Fio_2_ ratio, and Paco_2_. Time-varying proxies of lung and brain injury severity, respectively, included use of a neuromuscular blocking agent (used to treat moderate/severe hypoxemic respiratory failure) and thiopental (Pentothal; used to treat refractory intracranial hypertension).

All time-varying confounders were measured on days 1, 3, and 7 of mechanical ventilation. Our assumptions regarding covariate relationships and the temporal sequence of confounder-exposure feedback loops are summarized in a directed acyclic graph (e-Fig 1). Further details on definitions and covariate handling procedures are provided in e-Table 1.

### Statistical Analyses

Descriptive characteristics were presented for the entire cohort and across strata defined by exposure status and number of days after initiation of mechanical ventilation. Continuous variables were summarized using means and SD or medians and interquartile range (IQR) as appropriate. Categorical variables were summarized using absolute values with percentages.

We evaluated the effect of LTVV on mortality using marginal structural models.^29^ Logistic regression was used to generate stabilized inverse probability of treatment weights for each Vt group on each day of mechanical ventilation.^30^^,^^31^ For patients who progressed to spontaneous ventilation, where Vt cannot be precisely regulated, we adjusted for ventilator mode in our weighted analyses. To mitigate selection bias introduced by censoring patients who developed ARDS or were discharged from the ICU, we generated stabilized inverse probability of censoring weights for these events. Weights were based on both time-fixed and time-varying factors. The combined cumulative weight (stabilized inverse probability of treatment weights and stabilized inverse probability of censoring weights) was truncated at the 99th percentile and incorporated into a weighted time-dependent Cox model.^32^ Point estimates therefore represent marginal hazard ratios (HRs) and describe the population-level average effect for those exclusively treated with LTVV (ie, Vt ≤ 8 mL/kg at all 3 time points) vs those who never received LTVV (ie, Vt > 8 mL/kg PBW at all 3 time points).^33^ Importantly, individual patients did not contribute to the analysis as members of fixed exposure groups. Instead, their contribution to the overall pseudo-population was weighted according to their probability of remaining adherent to a given Vt strategy over time, conditional on current and past covariate history. In addition, by censoring patients on the basis of ARDS development and ICU discharge, our effect estimates reflect those from a pseudo-population where neither of these events occur.

Robust SEs were used to calculate 95% CIs. Missing data were handled using multiple imputation by chained equations, with 30 imputed data sets, and pooled using Rubin’s rules.^34^ Cumulative incidence curves were plotted using an average of the weighted cumulative incidences in the first 60 days across imputed data sets.

### Subgroup Analyses

We conducted prespecified subgroup analyses to explore potential effect modification of LTVV based on age (> 65 vs ≤ 65 years), brain injury severity (defined by admission GCS < 8 vs ≥ 8), baseline hypoxemic respiratory failure (Pao_2_/Fio_2_ ≤ 300 vs > 300), and ABI etiology (traumatic vs nontraumatic). We did not repeat subgroup analyses for different ABI subtypes because of the small sample sizes in several groups.

### Sensitivity Analyses

We performed several sensitivity analyses. First, to determine whether results were influenced by our definition of LTVV, we repeated the analysis using a series of incrementally lower Vt thresholds for the main exposure (7.5, 7, 6.5, and 6 mL/kg PBW). Second, we excluded participants who had no Vt data at any of the 3 time points and thus required imputation of their entire exposure history. Third, to detect potential model misspecification, we repeated the primary analysis using a flexible estimator (the “Super Learner”) to generate treatment weights.^35^ Fourth, we applied a negative control outcome to explore the impact of unmeasured confounding.^36^ For this analysis, we refit the models using ventilator-associated pneumonia (VAP) as our end point, anticipating a null association if the impact of unmeasured confounding was minimal.

Additional details of the statistical analyses are in e-Appendix 2. Sample size was determined in the original ENIO study, and no additional power calculations for this secondary analysis were done.^37^ All analyses were performed with R version 4.0.2 (R Foundation for Statistical Computing).

## Results

From 1,512 patients in the parent study, 2 individuals were excluded for having completely missing Vt and follow-up data, resulting in an analytic cohort of 1,510 patients. Mean age was 52 years (SD 18), 513 patients (34.0%) were female, and the most common etiologies of ABI were traumatic brain injury (n = 726; 48.1%) and intracranial hemorrhage (n = 521; 34.5%). Median GCS before endotracheal intubation was 7 (IQR, 5-9). Mean follow-up duration was 15 days (SD 13). Additional baseline characteristics are presented in Table 1.

**Table 1:** Baseline Characteristics

	All	Day 1 Vt ≤ 8	Day 1 Vt > 8	Absolute SMD
Characteristic	(n = 1,510)	(n = 1,095)^a^	(n = 330)^a^	
Mean age, y	51.7 (18.2)	50.5 (18.4)	55.4 (16.7)	0.28
Data missing	12	6	5	NA
Sex (female)	513 (34%)	294 (27%)	192 (58%)	0.67
Median BMI, kg/m^2^	25.58 (22.85-28.73)	25.26 (22.68-28.04)	26.67 (23.44-30.85)	0.38
Data missing	46	NA	NA	NA
Etiology of ABI				
Traumatic brain injury	726 (48%)	564 (52%)	121 (37%)	0.30
Subarachnoid aneurysmal hemorrhage	269 (18%)	190 (17%)	67 (20%)	0.08
Intracranial hemorrhage	521 (35%)	371 (34%)	120 (36%)	0.05
Ischemic stroke	141 (9.3%)	99 (9.0%)	30 (9.1%)	0.00
CNS infection	74 (4.9%)	54 (4.9%)	19 (5.8%)	0.04
Brain tumor	72 (4.8%)	49 (4.5%)	20 (6.1%)	0.07
Other	33 (2.2%)	21 (1.9%)	8 (2.4%)	0.04
Data missing	2	1	1	NA
Median GCS score before intubation (IQR)	7.00 (5.00-9.00)	7.00 (5.00-8.50)	7.00 (5.00-9.00)	0.04
Health status before admission				
Pulmonary disease	51 (3.4%)	33 (3.0%)	15 (4.6%)	0.08
Data missing	1	0	1	NA
Hypertension	450 (30%)	301 (27%)	117 (36%)	0.17
Data missing	1	0	1	NA
Heart failure	44 (2.9%)	31 (2.8%)	10 (3.0%)	0.01
Data missing	1	0	1	NA
Active smoking	330 (22%)	250 (23%)	68 (21%)	0.05
Data missing	9	3	4	NA
Diabetes mellitus	183 (12%)	119 (11%)	55 (17%)	0.17
Data missing	1	0	1	NA
History of malignancy	68 (4.5%)	51 (4.7%)	12 (3.6%)	0.05
Data missing	1	0	1	NA
Ventilatory parameters on admission				
Mean Pao_2_/Fio_2_ ratio	331.00 (150.00)	334.00 (157.00)	317.00 (130.00)	0.12
Data missing	36	13	3	NA
Mean respiratory rate	17.00 (4.00)	17.00 (4.00)	16.00 (3.00)	0.41
Data missing	24	NA	NA	NA
Mean Paco_2_	38.00 (17.00)	39.00 (18.00)	37.00 (13.00)	0.11
Data missing	34	13	3	NA
Mean plateau pressure	16.50 (4.40)	16.30 (4.20)	16.80 (4.70)	0.11
Data missing	255	166	43	NA
Mean V_T_	7.29 (1.22)	6.77 (0.71)	9.01 (1.00)	2.6
Data missing	85	NA	NA	NA

Data are presented as No. (%) or mean (SD) unless otherwise indicated. ABI = acute brain injury; CNS = central nervous system; GCS = Glasgow Coma Scale; IQR = interquartile range; NA = not applicable; SMD = standardized mean difference; Vt = tidal volume.

a Excludes 85 participants with missing Vt data on day 1.

**Figure 1:**
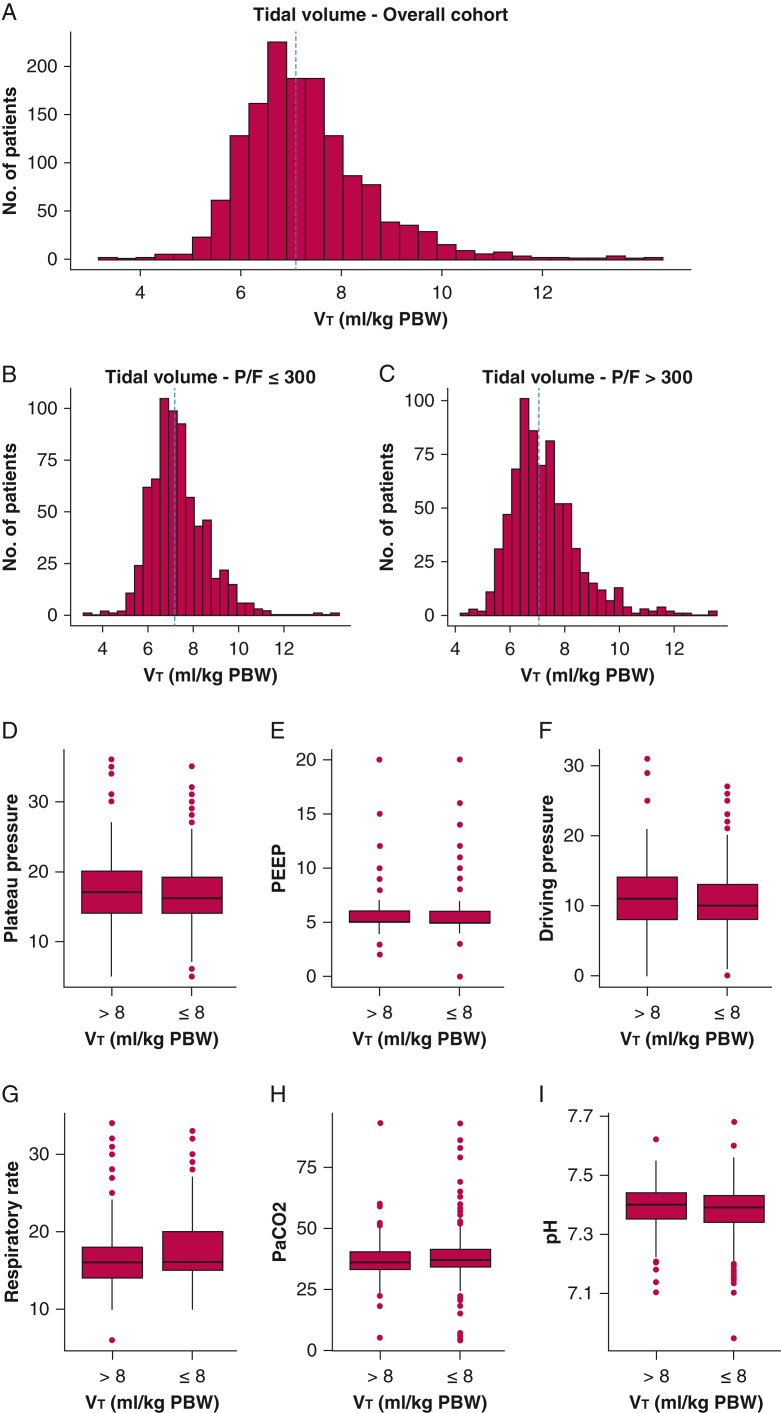
Distribution of mechanical ventilation and gas exchange parameters on day 1 of mechanical ventilation. A-C, Histograms for tidal volume (in mL/kg PBW) in the overall cohort (A) and stratified by P/F ratio (B and C). The vertical blue line in each of the panels represents the median tidal volume. D-I, Boxplots for additional ventilatory parameters stratified by day 1 tidal volume (in mL/kg PBW). PBW = predicted body weight; PEEP = positive end-expiratory pressure; P/F = ratio of arterial partial pressure of oxygen to Fio_2_; Vt = tidal volume.

The most common mode of ventilation at all 3 time points was volume assist/control (e-Table 2). On day 1 of invasive mechanical ventilation, 1,095 patients (76.8%) received LTVV, compared with 887 (72.6%) of those still intubated on day 3, and 565 (68.1%) of those still intubated on day 7. Mean Vt in the LTVV and control groups, respectively, were 6.8 mL/kg (SD 0.7) and 9.0 mL/kg (SD 1.0) on day 1; 6.7 mL/kg (SD 0.7) and 9.3 mL/kg (SD 1.2) on day 3; and 6.8 mL/kg (SD 0.8) and 9.2 mL/kg (SD 1.2) on day 7 (e-Fig 2). Characteristics were similar between patients who had missing Vt data at any time point and those with complete data (e-Table 3). Mean P_plat_ in the LTVV and control groups were, respectively, 16 cm H_2_O (SD 4) and 17 cm H_2_O (SD 5) on day 1; 17 cm H_2_O (SD 4) and 16 cm H_2_O (SD 5) on day 3; and 17 cm H_2_O (SD 5) and 16 cm H_2_O (SD 5) on day 7 (Fig 1, e-Fig 3). There was no difference between groups with respect to P_plat_, PEEP, driving pressure, Paco_2_, and pH on day 1 (Fig 1). Stratified comparisons for these variables on days 3 and 7 are shown in e-Figure 3. Patients at risk of high ICP, as judged by the presence of an external ventricular drain or intraparenchymal probe, had similar Vt, Paco_2_, and pH compared with patients without a monitor (e-Fig 4).

**Figure 2:**
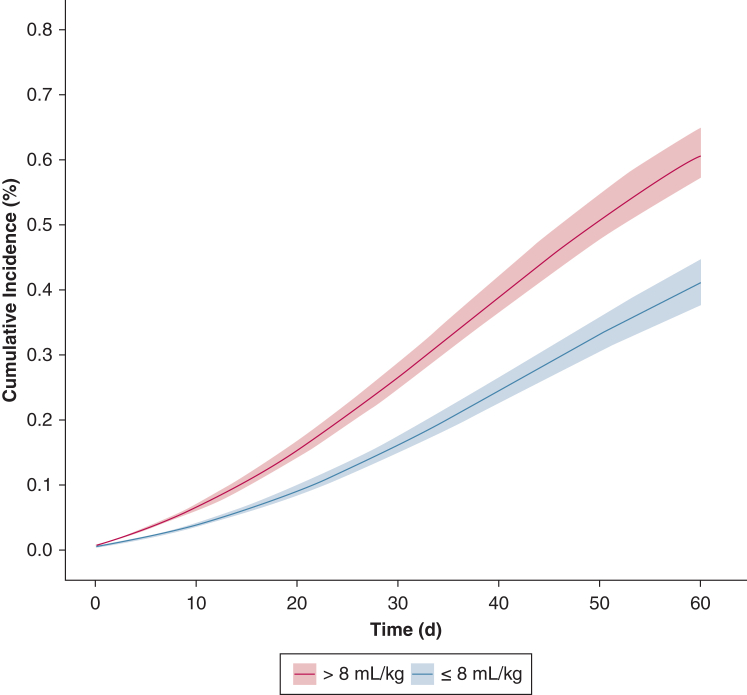
Cumulative incidence of ICU mortality between patients treated with low-tidal-volume ventilation vs those who received tidal volume ventilation > 8 mL/kg predicted body weight. Survival probabilities were estimated for each imputed data set (n = 30) and averaged across data sets. Shaded areas represent 95% confidence intervals.

**Figure 3:**
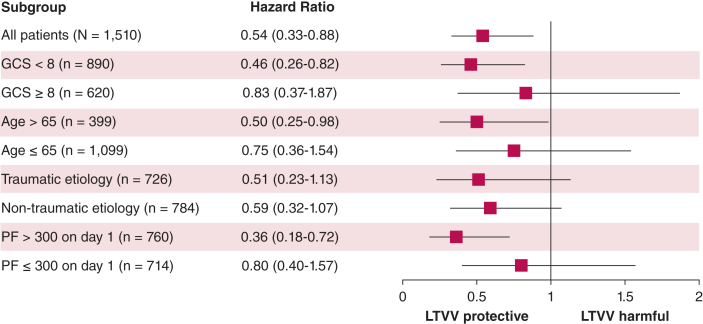
Forest plot for the main analysis (top row) and subgroup analyses (additional rows). In each analysis, point estimates represent the hazard ratio for ICU mortality at 60 days in patients who received LTVV vs those who received tidal volume ventilation > 8 mL/kg predicted body weight. Horizontal lines indicate 95% CIs. GCS = Glasgow Coma Scale; LTVV = low-tidal-volume ventilation; PF = ratio of arterial partial pressure of oxygen to Fio_2_.

By day 60, 122 patients (8.1%) died. A total of 1,185 (78.5%) were censored, of whom 137 (9.2%) developed ARDS during ICU admission and the remaining were discharged from the ICU before 60 days. In adjusted analyses, the cumulative incidence of mortality at 60 days in the pseudo-population of patients who received LTVV over the first 7 days of mechanical ventilation was 40.2% (95% CI, 19.2%-61.1%), compared with 59.7% (95% CI, 44.0%-75.4%) in patients who received Vt > 8 mL/kg PBW (Fig 2). The marginal HR for mortality was 0.54 (95% CI, 0.33-0.88) (Fig 3). Results were consistent in subgroups of patients with GCS < 8, age > 65 years, and baseline Pao_2_/Fio_2_ > 300. For the remaining subgroups, point estimates were in a similar direction to the main analysis but with considerable imprecision as reflected by wide CIs (Fig 3).

Sensitivity analyses are presented in Table 2. Using the Super Learner and excluding patients with missing Vt yielded similar results to the primary analysis. When LTVV was evaluated using lower thresholds for Vt, the association with 60-day mortality was indeterminate across all analyses, as reflected by wide CIs. In the analysis of a negative control outcome, LTVV was not associated with nosocomial VAP (HR, 1.14; 95% CI, 0.72-1.81).

**Table 2:** Sensitivity Analyses

Analysis	HR (95% CI)
Super Learner (n = 1,510)	0.52 (0.27-1.00)
Excluding participants without tidal volumes at any time point (n = 1,448)	0.53 (0.33-0.87)
LTVV defined as ≤ 7.5 mL/kg PBW (n = 1,510)	0.73 (0.46-1.16)
LTVV defined as ≤ 7 mL/kg PBW (n = 1,510)	0.87 (0.54-1.41)
LTVV defined as ≤ 6.5 mL/kg PBW (n = 1,510)	0.73 (0.38-1.38)
LTVV defined as ≤ 6 mL/kg PBW (n = 1,510)	1.12 (0.39-3.19)
Nosocomial VAP (n = 1,510)	1.14 (0.72-1.81)

HR = hazard ratio; LTVV = low-tidal-volume ventilation; PBW = predicted body weight; VAP = ventilator-associated pneumonia.

## Discussion

In the present analysis, we found that LTVV with Vt ≤ 8 mL/kg PBW over the first 7 days of mechanical ventilation was associated with lower ICU mortality in patients with ABI, compared with Vt > 8 mL/kg PBW. There was no convincing heterogeneity of treatment effect in multiple subgroup analyses. When using Vt thresholds below 8 mL/kg PBW to define LTVV, the association between ventilatory strategy and mortality was less clear. Taken together, these results cautiously support a clinical strategy limiting exposure to high Vt (> 8 mL/kg PBW) in patients with ABI, consistent with data from other critically ill populations.^3^^,^^7^^,^^8^

In our descriptive analysis, we found that exposure to Vt > 8 mL/kg PBW remains common among patients with ABI. Of the patients in our cohort, 23% had Vt above this threshold at ICU admission, which might reflect concerns about the effect of LTVV on ICP mediated by hypercarbia. Nevertheless, these concerns must be weighed against the risk of propagating VILI from high Vt in a population that is already highly vulnerable to lung injury from sympathetic activation, proinflammatory cytokine release, and immune dysregulation caused by ABI.^38^^,^^39^ Current evidence in patients with ABI does not definitively answer the question of when, and in whom, LTVV should be used. As a result, ventilation strategies in this population remain highly variable.^10^^,^^40^ In a before-after study from 2 ICUs in France, implementation of a ventilatory bundle that incorporated use of Vt at 6 to 8 mL/kg PBW was associated with a shorter duration of mechanical ventilation and more ICU-free days at day 90; however, findings could have been explained by other aspects of the bundle, such as early extubation.^41^ In 2 observational studies, high Vt was associated with higher risk of developing ARDS, but results may have been susceptible to time-dependent confounding bias.^9^^,^^42^ In a consensus guideline, all 4 recommendations pertaining to lung-protective ventilation in patients with ABI were supported by no evidence, and good practice statements to inform recommendations relied on data from non-ABI populations.^10^

The Lung-Protective Mechanical Ventilation in Patients With Severe Acute Brain Injury (PROLABI) trial, published in 2024, found that a protective ventilation strategy (defined as Vt = 6 mL/kg PBW and PEEP 4 cm H_2_O) led to worse short-term outcomes (defined as a primary composite end point of death, ventilator dependence, or ARDS by day 28) and worse 6-month neurologic outcomes compared with conventional ventilation (defined as Vt ≥ 8 mL/kg PBW and PEEP 4 cm H_2_O).^13^ Caveats of this trial include underpowering, low interrater agreement for ARDS diagnosis, and lack of a clear physiologic rationale for the observed differences.^14^ In contrast, we found a significant association between LTVV and lower ICU mortality, which could reflect our use of a higher Vt threshold (≤ 8 mL/kg PBW) to define LTVV. Findings from our study align with evidence from broader ICU populations^3^^,^^8^ and support best-practice recommendations for mechanical ventilation in ABI guidelines.^10^ Nevertheless, given conflicting results in the current body of evidence, additional high-quality trials are essential to determining the optimal Vt strategy, while addressing potential risks related to hypercarbia and ICP and exploring effects in different ABI subgroups where clinical responses to LTVV may vary.

In sensitivity analyses, we found that associations between Vt and ICU mortality were indeterminate at thresholds below 8 mL/kg PBW. Findings from these analyses cannot be used to support or refute a true protective effect of further reductions in Vt on mortality. Conceptually, it is also possible that the increase in respiratory rate required to offset hypercarbia with Vt reduction may increase mechanical power, which has been associated with increased mortality in critically ill patients, including those with ABI.^10^^,^^43^ In this context, additional mediators of VILI beyond Vt (including driving pressure and mechanical power) warrant further investigation as potential targets to improve clinical outcomes.

This study has important strengths. We demonstrate the application of a marginal structural model to evaluate LTVV in patients with ABI during a clinically relevant period of high acuity.^29^^,^^30^ Marginal structure models capture the complex interplay between dynamic treatments and confounders and may be especially useful in critical care research where treatments are frequently manipulated in response to changing illness characteristics.^44^ Using traditional regression methods in this setting can lead to bias.^29^^,^^30^ We also included a novel Super Learner method to flexibly estimate the probability of receiving LTVV.^35^ This data-adaptive approach to exposure assignment is robust to model misspecification. Finally, we used data from a large study of patients treated across 73 ICUs in 18 countries, enhancing the generalizability of our findings.

This study has limitations. First, our primary outcome reflects mortality in a pseudo-population in which all patients remained in the ICU up to 60 days and no patients developed ARDS. This restriction was necessary because the primary study did not record the day of ARDS onset or death after ICU discharge, and therefore did not support time-varying modeling of these events. While our approach limits generalizability to the broader ABI population, it enabled valid estimation of the causal effect of sustained LTVV under well-defined and transparent assumptions. Second, we could not address confounding on a more granular timescale (eg, minutes or hours) because time-varying confounders were available only on days 1, 3, and 7 of mechanical ventilation. Third, unmeasured confounding is likely present because the parent study did not capture all variables clinicians would typically consider when manipulating Vt, such as ICP.^19^ In a descriptive analysis stratified by the presence or absence of an ICP monitor, we found that Vt and other parameters of mechanical ventilation were similar between groups. We also adjusted for the use of Pentothal, which is typically used to treat high ICP. Nevertheless, time-varying ICP values were not available, and both the presence of an invasive monitor and Pentothal use are imperfect surrogates of ICP risk.^45^ However, the association between LTVV and a negative control outcome (VAP) was not significant, suggesting that our findings are robust to residual confounding. Fourth, some subgroup and sensitivity analyses demonstrated wide CIs, likely resulting from small sample sizes or positivity violations as few patients remained adherent to their assigned strategy at all 3 time points. Fifth, we did not repeat subgroup analyses for each ABI condition because of small sample sizes in several groups. It is therefore possible that mortality within subgroups could differ from the overall cohort effect, potentially due to unique pathophysiologic responses that may influence the efficacy of LTVV by underlying diagnosis. Sixth, models were fitted for a single outcome (ICU mortality), and additional benefits of LTVV could exist for other end points, such as long-term neurologic outcomes.

## Interpretation

In this predominantly non-ARDS cohort of 1,510 patients with ABI, a strategy of LTVV during the first 7 days of invasive mechanical ventilation was associated with lower ICU mortality up to 60 days compared with ventilation with Vt > 8 mL/kg PBW. Additional evidence from adequately powered randomized trials is necessary to establish whether sustained interventions to limit Vt could reduce mortality in this population. Future studies should also explore effects on neurologic outcomes and safety end points including ICP.

## Funding/Support

J. F. D. is funded by research scholarships from the 10.13039/501100000024Canadian Institutes of Health Research (Vanier Canada Graduate Scholarship) and the Department of Surgery at the University of Toronto. M. U. is supported by an IDCCM Scholar Award from the Interdepartmental Division of Critical Care Medicine, 10.13039/501100003579University of Toronto. S. W. receives support from the 10.13039/100000002National Institutes of Health. S. T. is funded by the Eliot Phillipson Clinician-Scientist Training Program at the University of Toronto, the Canadian Institutes of Health Research (Doctoral Award), and an IDCCM Scholar Award from the Interdepartmental Division of Critical Care Medicine, University of Toronto.

## Financial/Nonfinancial Disclosures

The authors have reported to *CHEST* the following**:** C. R. received fees as speaker for Edwards and BT. R. D. S. received consulting fees from Ceribell Inc. and speaker honoraria from the University of Texas MD Anderson Cancer Center. R. C. received consulting fees from Viatris. S. T. received speaker fees from the Mechanical Ventilation Symposium, Interdepartmental Division of Critical Care Medicine, University of Toronto. None declared (J. F. D., D. M. H., M. U., K. L., S. W., and V. A. McC.).

## Supplementary Data

e-Online Data

e-Online Data

Audio
