# Supplementary material for: Low-Tidal-Volume Ventilation and Mortality in Patients With Acute Brain Injury: A Secondary Analysis of an International Observational Study
Source: Chest. 2025 Jul 8;168(5):1141–51. doi: 10.1016/j.chest.2025.06.042 (PMC12833485; doi:10.1016/j.chest.2025.06.042)
Supplement: e-Online Data [file mmc1.docx]

**Supplementary Material**

Table of Contents

[e-Appendix 1: STROBE Checklist 2](#_Toc201793909)

[e-Figure 1: Directed Acyclic Graph (DAG) 5](#_Toc201793910)

[e-Table 1: Key Definitions 6](#_Toc201793911)

[e-Appendix 2: Expanded Statistical Methods 7](#_Toc201793912)

[e-Table 2: Modes of Ventilation 9](#_Toc201793913)

[e-Figure 2: Distribution of Tidal Volumes 10](#_Toc201793914)

[e-Table 3: Characteristics of Patients with Missing Tidal Volume 11](#_Toc201793915)

[e-Figure 3: Ventilator Data by Tidal Volume Strata 12](#_Toc201793916)

[e-Figure 4: Gas Exchange Variables by Strata of Tidal Volume and ICP Monitors 14](#_Toc201793917)

[References 16](#_Toc201793918)

# **e-Appendix 1: STROBE Checklist**

|  | Item No | Recommendation | Page  No |
| --- | --- | --- | --- |
| **Title and abstract** | 1 | (*a*) Indicate the study’s design with a commonly used term in the title or the abstract | 1, 3 |
|  |  | (*b*) Provide in the abstract an informative and balanced summary of what was done and what was found | 3 |
| Introduction | | | |
| Background/rationale | 2 | Explain the scientific background and rationale for the investigation being reported | 4 |
| Objectives | 3 | State specific objectives, including any prespecified hypotheses | 5 |
| Methods | | | |
| Study design | 4 | Present key elements of study design early in the paper | 6 |
| Setting | 5 | Describe the setting, locations, and relevant dates, including periods of recruitment, exposure, follow-up, and data collection | 6 |
| Participants | 6 | (*a*) *Cohort study*—Give the eligibility criteria, and the sources and methods of selection of participants. Describe methods of follow-up  *Case-control study*—Give the eligibility criteria, and the sources and methods of case ascertainment and control selection. Give the rationale for the choice of cases and controls  *Cross-sectional study*—Give the eligibility criteria, and the sources and methods of selection of participants | 6 |
|  |  | (*b*) *Cohort study*—For matched studies, give matching criteria and number of exposed and unexposed  *Case-control study*—For matched studies, give matching criteria and the number of controls per case | N/A |
| Variables | 7 | Clearly define all outcomes, exposures, predictors, potential confounders, and effect modifiers. Give diagnostic criteria, if applicable | 6-8 |
| Data sources/ measurement | 8* | For each variable of interest, give sources of data and details of methods of assessment (measurement). Describe comparability of assessment methods if there is more than one group | Supp |
| Bias | 9 | Describe any efforts to address potential sources of bias | 9 |
| Study size | 10 | Explain how the study size was arrived at | 10 |
| Quantitative variables | 11 | Explain how quantitative variables were handled in the analyses. If applicable, describe which groupings were chosen and why | Supp |
| Statistical methods | 12 | (*a*) Describe all statistical methods, including those used to control for confounding | 8, 9,  Supp |
|  |  | (*b*) Describe any methods used to examine subgroups and interactions | 9 |
|  |  | (*c*) Explain how missing data were addressed | 9, Supp |
|  |  | (*d*) *Cohort study*—If applicable, explain how loss to follow-up was addressed  *Case-control study*—If applicable, explain how matching of cases and controls was addressed  *Cross-sectional study*—If applicable, describe analytical methods taking account of sampling strategy | 7 |
|  |  | (*e*) Describe any sensitivity analyses | 9 |

Continued on next page

| Results | | | |
| --- | --- | --- | --- |
| Participants | 13* | (a) Report numbers of individuals at each stage of study—eg numbers potentially eligible, examined for eligibility, confirmed eligible, included in the study, completing follow-up, and analysed | 11 |
|  |  | (b) Give reasons for non-participation at each stage | N/A |
|  |  | (c) Consider use of a flow diagram | - |
| Descriptive data | 14* | (a) Give characteristics of study participants (eg demographic, clinical, social) and information on exposures and potential confounders | 11, Table 1 |
|  |  | (b) Indicate number of participants with missing data for each variable of interest | Table 1 |
|  |  | (c) *Cohort study*—Summarise follow-up time (eg, average and total amount) | 11 |
| Outcome data | 15* | *Cohort study*—Report numbers of outcome events or summary measures over time | 11 |
|  |  | *Case-control study—*Report numbers in each exposure category, or summary measures of exposure |  |
|  |  | *Cross-sectional study—*Report numbers of outcome events or summary measures |  |
| Main results | 16 | (*a*) Give unadjusted estimates and, if applicable, confounder-adjusted estimates and their precision (eg, 95% confidence interval). Make clear which confounders were adjusted for and why they were included | 11 |
|  |  | (*b*) Report category boundaries when continuous variables were categorized | 6, 7 |
|  |  | (*c*) If relevant, consider translating estimates of relative risk into absolute risk for a meaningful time period | 11, 12 |
| Other analyses | 17 | Report other analyses done—eg analyses of subgroups and interactions, and sensitivity analyses | 12 |
| Discussion | | | |
| Key results | 18 | Summarise key results with reference to study objectives | 13 |
| Limitations | 19 | Discuss limitations of the study, taking into account sources of potential bias or imprecision. Discuss both direction and magnitude of any potential bias | 15, 16 |
| Interpretation | 20 | Give a cautious overall interpretation of results considering objectives, limitations, multiplicity of analyses, results from similar studies, and other relevant evidence | 13, 14 |
| Generalisability | 21 | Discuss the generalisability (external validity) of the study results | 15 |
| Other information | | | |
| Funding | 22 | Give the source of funding and the role of the funders for the present study and, if applicable, for the original study on which the present article is based | 1 |

Page numbers refer to the document at the time of manuscript submission

# **e-Figure 1: Directed Acyclic Graph (DAG)**

Time-fixed covariates and time-dependent covariates were entered into the inverse probability models for treatment assignment. **Time-fixed covariates** included age, sex, brain injury etiology (traumatic brain injury aneurysmal, subarachnoid haemorrhage, intracranial haemorrhage, ischemic stroke, brain tumour, central nervous system infection, or other), lowest Glasgow Coma Scale score before intubation, and medical comorbidities (chronic obstructive pulmonary disease, heart failure, smoking status). **Time-dependent covariates** included ventilatory setting (controlled vs. assisted), P/F ratio, plateau pressure, respiratory rate, PaCO_2_, and administration of neuromuscular blockers or pentothal.

**
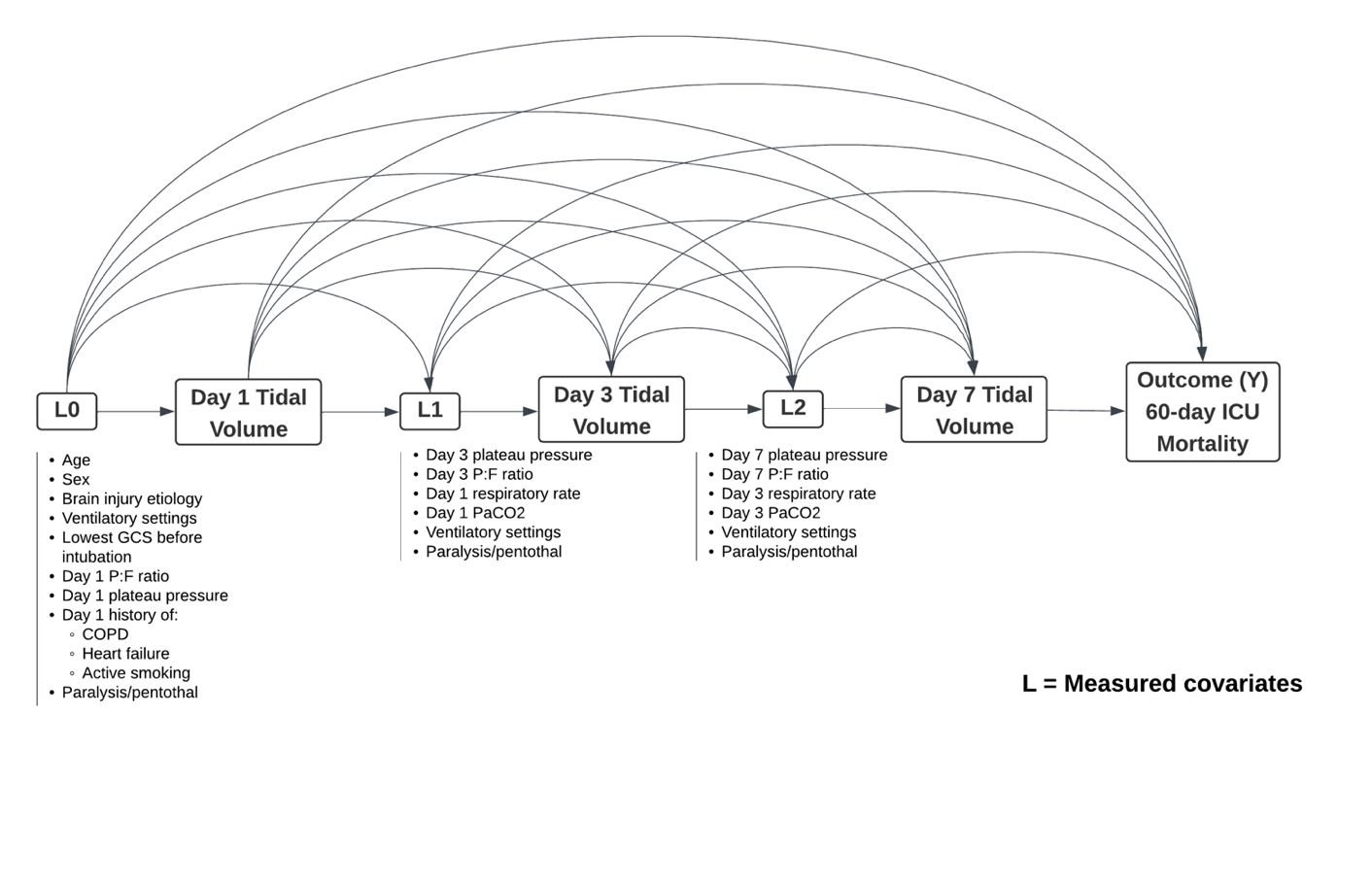
**

# **e-Table 1: Key Definitions**

| **Variable** | **Definition** | **Timing of Measurement** |
| --- | --- | --- |
| LTVV | Mechanical ventilation strategy using a tidal volume ≤8 mL/kg PBW | Days 1, 3, and 7 |
| Control group | Mechanical ventilation strategy using a tidal volume >8 mL/kg PBW | Days 1, 3, and 7 |
| Age | In years | Day 1 |
| Sex | - Male - Female | Day 1 |
| Acute brain injury etiology | - Traumatic brain injury - Subarachnoid aneurysmal haemorrhage - Intracranial haemorrhage - Ischemic stroke - Brain tumour - Central nervous system infection - Other | Day 1 |
| GCS | Lowest GCS score prior to initiation of mechanical ventilation | Day 1 |
| Medical comorbidities | - Pulmonary disease - Heart failure - Active smoking | Day 1 |
| PaO_2_/FiO_2_ | Ratio of the partial pressure of oxygen to the fraction of inspired oxygen concentration | Days 1, 3, and 7 |
| P_plat_ | Plateau pressure | Days 1, 3, and 7 |
| Respiratory Rate | In breaths/minute | Days 1, 3, and 7 |
| PaCO_2_ | Partial pressure of arterial carbon dioxide | Days 1, 3, and 7 |
| Ventilatory Mode | - Controlled - Assisted | Days 1, 3, and 7 |
| Sedation/Paralysis | Sedation using pentothal or paralysis using a neuromuscular blocking agent (used as proxies of brain and lung injury severity, respectively) | Days 1, 3, and 7 |

Abbreviations: GCS, Glasgow Coma Scale; LTVV, low tidal volume ventilation; PBW, predicted body weight

# **e-Appendix 2: Expanded Statistical Methods**

Marginal Structural Models

We used marginal structural Cox models with inverse probability weighting to estimate the causal effect of LTVV on 60-day mortality. Unlike standard regression approaches—including Cox models with time-varying covariates—this method appropriately adjusts for time-varying confounders affected by prior exposure.

Here we describe, in more detail, how we fit the marginal structural models. First, we generated stabilized inverse probability of treatment weights (sIPTWs) for each day of follow-up (i.e., days 1, 3 and 7 following initiation of mechanical ventilation) using multivariable logistic regression.^1,2^ For patients who were extubated after day 1, we imputed their last recorded V_T_ at days 3 and 7, similar to a last-observation carried forward approach. For example, if a patient was extubated on day 2, we imputed their exposure on days 3 and 7 with their exposure from day 1. Weights were based on both time-fixed and time-varying factors that clinicians might consider when deciding which tidal volume strategy a patient should receive (see Directed Acyclic Graph). We also used stabilized inverse probability of censoring weights (sIPCW) to mitigate selection bias introduced by censoring patients who developed ARDS or were discharged from ICU. Censoring weights were generated for each day of follow-up using multivariable logistic regression, adjusted for time-fixed and time-dependent covariates.

Next, the combined cumulative weight (i.e., sIPTW multiplied by sIPCW) was truncated at the 99^th^ percentile and incorporated into a weighted time-dependent Cox model.^3^ In this model, we specified the comparison to be patients who remained adherent to their original exposure on day 1. Therefore, the overall analysis compared patients who received LTVV on days 1, 3, and 7 versus those who never received LTVV (at the same 3 timepoints). Point estimates represent marginal hazard ratios (HRs) and describe the average treatment effect of LTVV under full adherence to the initially assigned treatment strategy. We used robust standard errors to calculate 95% confidence intervals (CIs). Cumulative incidence curves were plotted using an average of the weighted cumulative incidences in the first 60 days across imputed datasets.

Super Learner

In a sensitivity analysis, we used the ‘Super Learner’ to estimate sIPTWs. The Super Learner is a data-adaptive estimator that involves cross-validation of a set of pre-specified algorithms and combines them in a weighted ensemble to flexibly model an endpoint (e.g., probability of receiving treatment).^4^ Compared to standard logistic regression, the Super Learner reduces bias in the presence of model misspecification (e.g., resulting from nonlinear effects and interactions).^5^ Our Super learner library consisted of the following supervised machine learning algorithms: random forests, neural networks, extreme gradient boosting, and K-Nearest Neighbours. The remaining steps were replicated from the main analysis.

Missing data

We explored patterns of missingness by comparing baseline covariates for subjects with and without missing exposure status. To account for missing data, we generated 30 imputed datasets using multiple imputation by chained equations.^6^ The imputation matrix integrated all variables relevant for our analyses (including failure time and survival status), as well as auxiliary variables related to ABI severity and co-interventions during ICU admission.^7^

|  | **LTVV adherent**  **N = 872** | **Partially adherent**  **N = 420** | **Control adherent**  **N = 218** |
| --- | --- | --- | --- |
| *Day 1* |  |  |  |
| Pressure assist | 78 (8.9%) | 49 (12%) | 41 (19%) |
| Pressure control | 152 (17%) | 85 (21%) | 56 (26%) |
| Spontaneous breathing | 7 (0.8%) | 16 (4.0%) | 2 (0.9%) |
| Volume assist | 635 (73%) | 253 (63%) | 118 (54%) |
| Unknown | 0 | 17 | 1 |
| *Day 3* |  |  |  |
| Pressure assist | 154 (21%) | 125 (31%) | 48 (28%) |
| Pressure control | 105 (14%) | 63 (16%) | 36 (21%) |
| Spontaneous breathing | 68 (9.2%) | 50 (13%) | 10 (5.7%) |
| Volume assist | 414 (56%) | 161 (40%) | 80 (46%) |
| Unknown | 131 | 21 | 44 |
| *Day 7* |  |  |  |
| Pressure assist | 126 (25%) | 142 (45%) | 50 (43%) |
| Pressure control | 62 (13%) | 32 (10%) | 23 (20%) |
| Spontaneous breathing | 81 (16%) | 67 (21%) | 11 (9.6%) |
| Volume assist | 227 (46%) | 74 (23%) | 31 (27%) |
| Unknown | 376 | 105 | 103 |
| ICU mortality | 62 (7.1%) | 37 (8.8%) | 23 (11%) |
| Censoring before 60 days | 799 (92%) | 376 (90%) | 194 (89%) |
| ARDS | 89 (10%) | 40 (9.6%) | 8 (3.7%) |
| Unknown | 10 | 4 | 3 |
| VAP | 103 (12%) | 61 (15%) | 19 (9.1%) |
| Unknown | 18 | 9 | 10 |

# **e-Table 2: Modes of Ventilation**

LTVV adherent means that patients received LTVV at all 3 timepoints or were censored / had missing data; partially adherent means patients received a combination of LTVV or tidal volumes>8ml/kg predicted body weight (PBW); control adherent means patients received tidal volumes>8ml/kg PBW at all 3 timepoints or were censored.

Abbreviations: ARDS, acute respiratory distress syndrome; LTVV, low tidal volume ventilation; VAP, ventilator associated pneumonia

**e-Figure 2: Distribution of Tidal Volumes**

Tidal volumes in mL/kg predicted body weight (PBW) are displayed for day 3 (top panel) and day 7 (bottom panel). The median tidal volume on each day is indicated by the vertical dotted red line.

**Day 3**


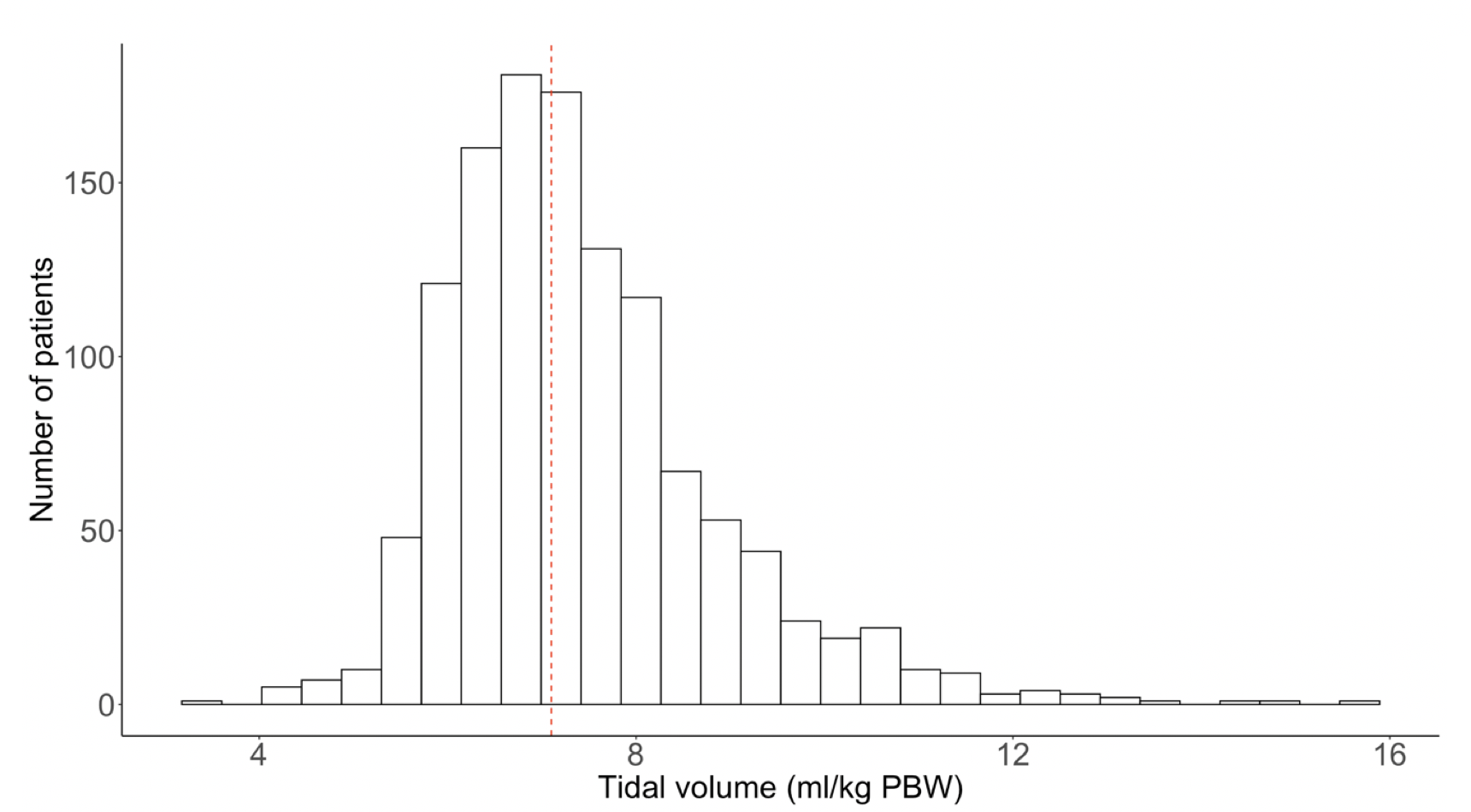


**Day 7**

**
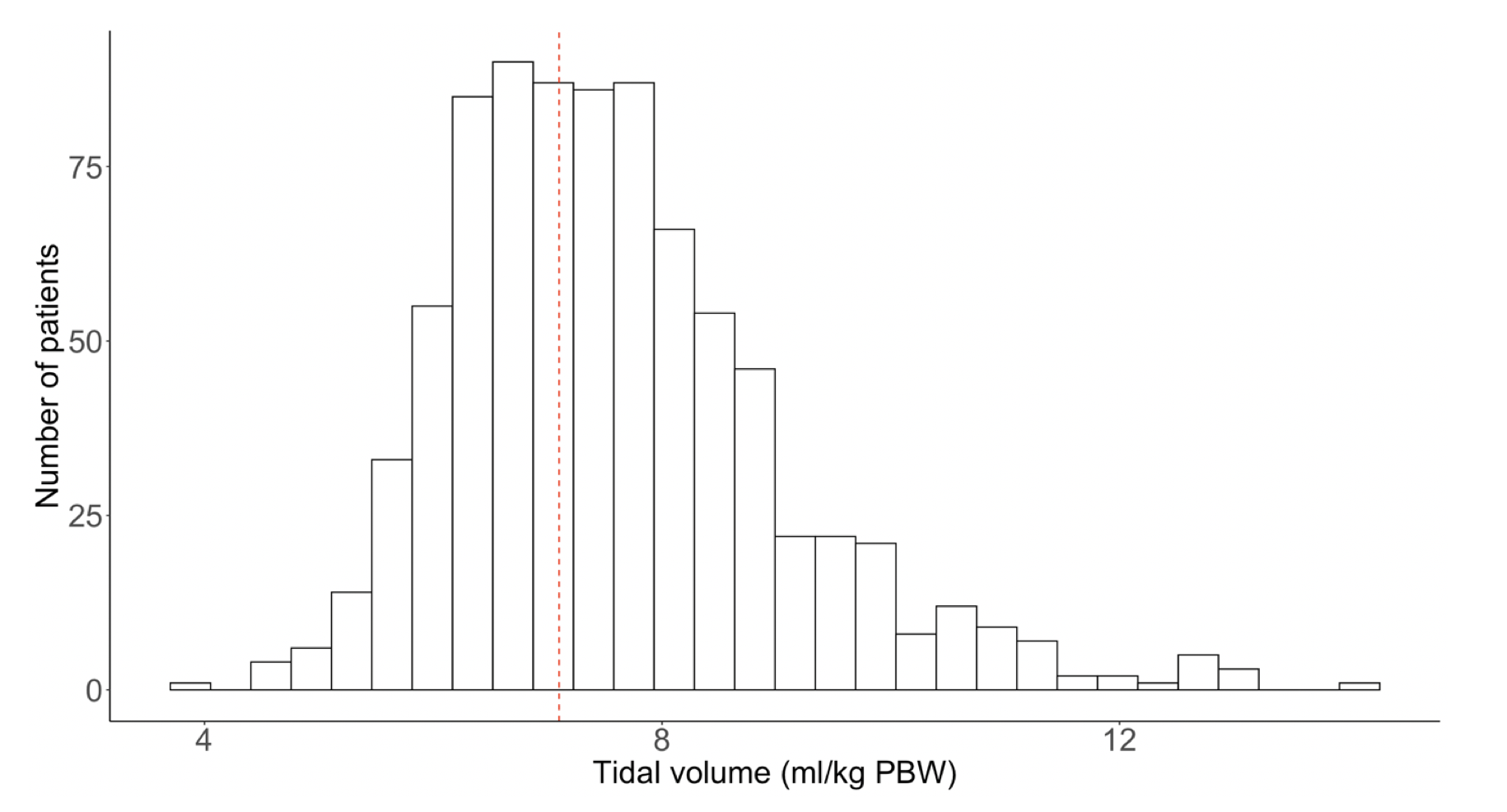
**

# **e-Table 3: Characteristics of Patients with Missing Tidal Volume**

| **Characteristic** | **No missing exposure,**  **N = 1,159** | **Missing exposure,**  **N = 351** | **Absolute**  **SMD** |
| --- | --- | --- | --- |
| Mean age (SD), in years | 51.63 (17.94) | 51.87 (19.03) | 0.01 |
| Female sex | 397 (34%) | 116 (33%) | 0.03 |
| Median BMI (SD), in kg/m^2^ | 25.61  (22.86, 28.73) | 25.47  (22.49, 28.40) | 0.05 |
| *Etiology of ABI* | | | |
| Traumatic brain injury | 558 (48%) | 168 (48%) | 0.01 |
| Subarachnoid hemorrhage | 212 (18%) | 57 (16%) | 0.05 |
| Intra-cranial hemorrhage | 387 (33%) | 134 (38%) | 0.10 |
| Ischemic stroke | 108 (9.3%) | 33 (9.4%) | 0.00 |
| Central nervous system infection | 60 (5.2%) | 14 (4.0%) | 0.06 |
| Brain tumour | 52 (4.5%) | 20 (5.7%) | 0.06 |
| Other | 22 (1.9%) | 11 (3.1%) | 0.08 |
| Median GCS before intubation (IQR) | 7.00  (5.00, 8.00) | 7.00  (5.50, 9.00) | 0.10 |
| *Health status prior to admission* | | | |
| Pulmonary disease | 40 (3.5%) | 11 (3.1%) | 0.02 |
| Hypertension | 337 (29%) | 113 (32%) | 0.07 |
| Heart failure | 34 (2.9%) | 10 (2.8%) | 0.01 |
| Active smoking | 271 (24%) | 59 (17%) | 0.16 |
| Diabetes mellitus | 144 (12%) | 39 (11%) | 0.04 |
| History of malignancy | 45 (3.9%) | 23 (6.6%) | 0.12 |
| *Ventilatory parameters on admission* | | | |
| Mean P/F ratio (SD) | 327.76 (152.98) | 342.26 (140.35) | 0.10 |
| Mean respiratory rate (SD) | 17.01 (3.70) | 16.87 (3.77) | 0.04 |
| Mean PaCO_2_ (SD) | 38.48 (17.44) | 37.69 (12.70) | 0.05 |
| Mean plateau pressure (SD) | 16.65 (4.31) | 15.70 (4.47) | 0.22 |
| *Exposures* | | | |
| LTVV on day 1 | 888 (77%) | 207 (78%) | 0.03 |
| LTVV on day 3 | 779 (72%) | 108 (74%) | 0.03 |
| LTVV on day 7 | 548 (68%) | 17 (77%) | 0.21 |
| *Endpoints* | | | |
| Censored | 911 (79%) | 274 (78%) | 0.01 |
| All-cause death | 97 (8.4%) | 25 (7.1%) | 0.05 |

Abbreviations: BMI, body mass index; GCS, Glasgow Coma Score; IQR, interquartile range; LTVV, low tidal volume ventilation; P/F ratio, ratio of partial pressure of oxygen to fraction of inspired oxygen concentration; SD, standard deviation; SMD, standardized mean difference

# **e-Figure 3: Ventilator Data by Tidal Volume Strata**

Ventilator variables are shown stratified by tidal volume (>8mL/kg predicted body weight [PBW]) vs ≤8 ml/kg PBW) on day 3 (current page) and day 7 (next page) of invasive mechanical ventilation. Panel A = plateau pressure; Panel B = positive end expiratory pressure (PEEP); Panel C = driving pressure; Panel D = respiratory rate; Panel E = PaCO_2_; Panel F = pH.

**Day 3**

**
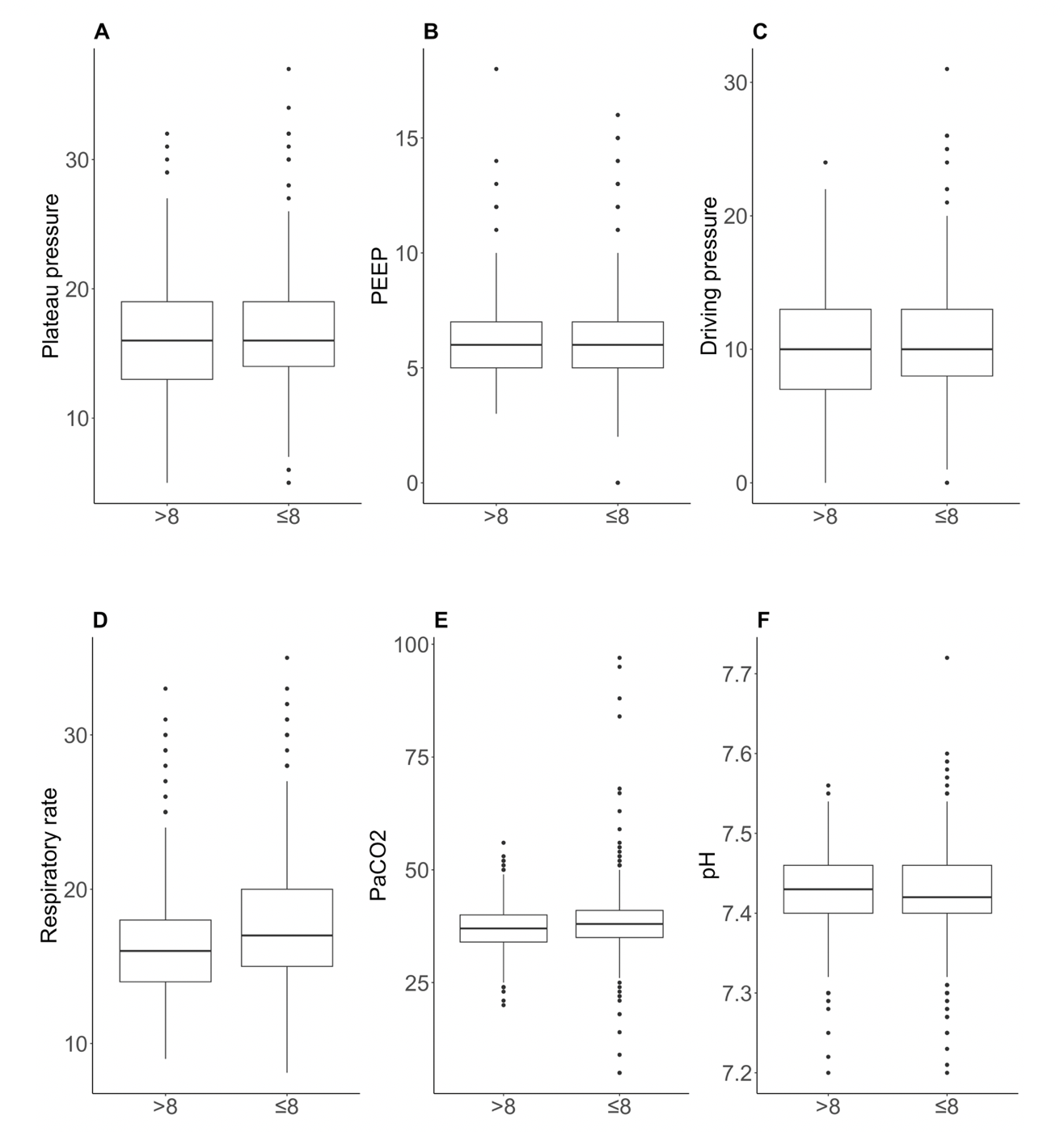
**

**Day 7**

**
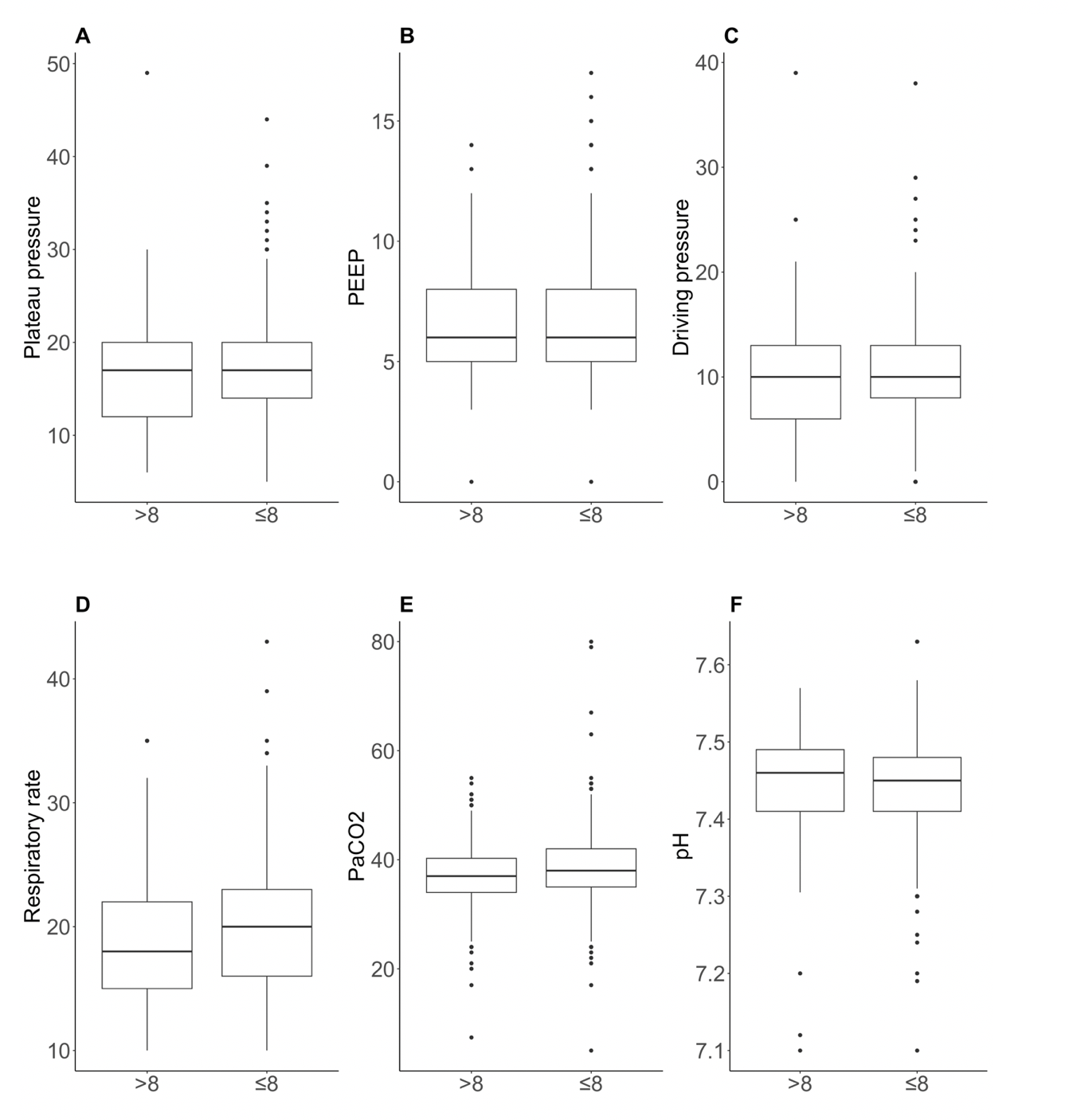
**

Abbreviations: PBW, predicted body weight; PEEP, positive end expiratory pressure

# **e-Figure 4: Gas Exchange Variables by Strata of Tidal Volume and ICP Monitors**

Data for PaCO_2_ and pH are shown stratified by tidal volume (≤ 8 ml/kg vs. > 8ml/kg PBW). On each day of mechanical ventilation, there was no difference in PaCO_2_ or pH between the tidal volume groups. To explore potential differences in gas exchange variables in patients with more severe forms of acute brain injury (ABI), data were further stratified according to presence or absence of an intracranial pressure probe (parenchymal monitor or external ventricular drain), which was used as a surrogate for higher brain injury severity. There was again no difference between PaCO­_2_ or pH when comparing similar tidal volume strategies across strata of patients with higher or lower ABI severity.

**
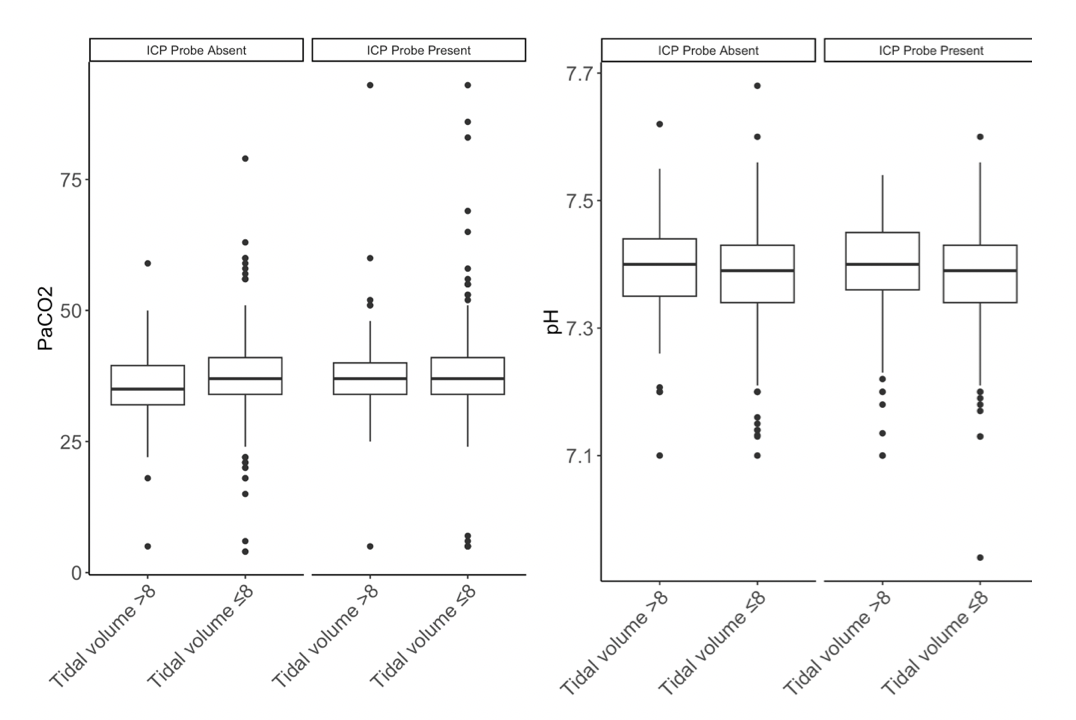
Day 1**

**Day 3**

**
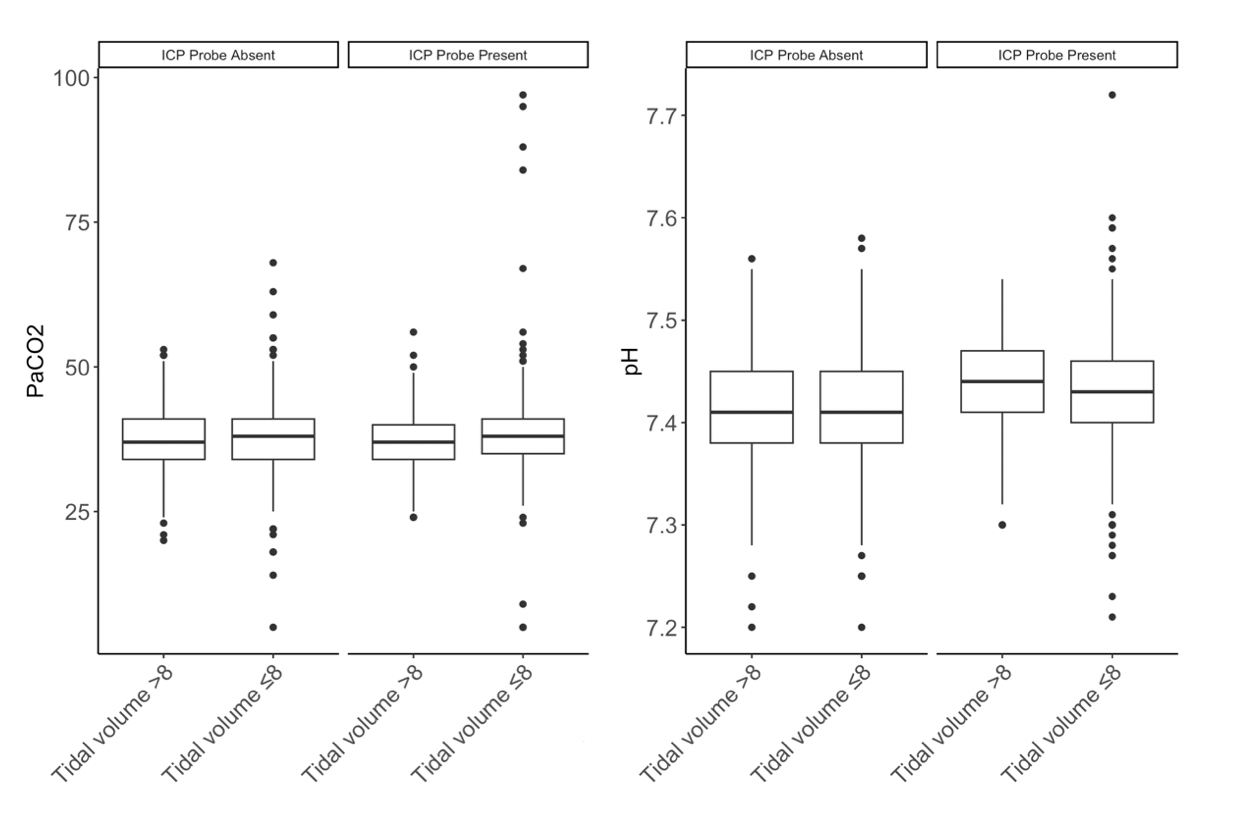
**

**Day 7**

**
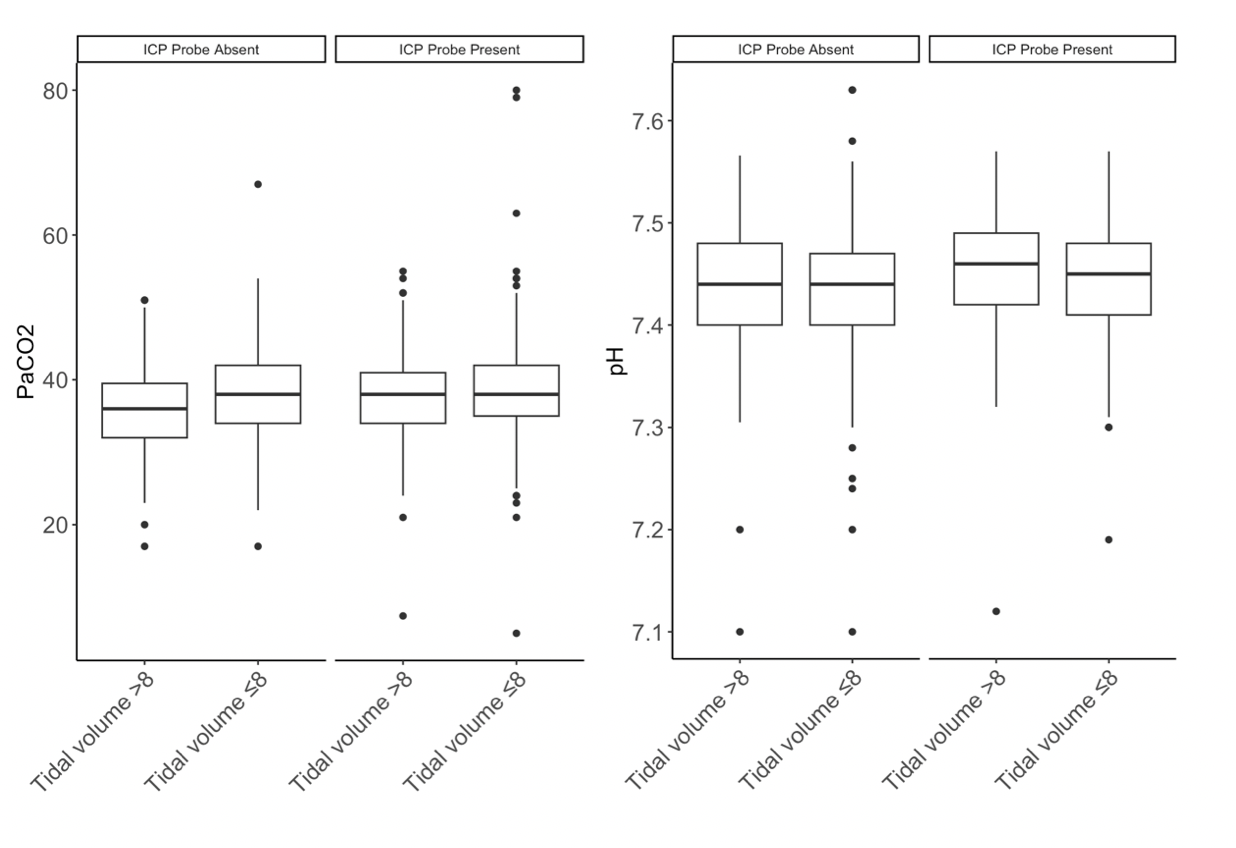
**

# **References**

1. Hernán MA. How to estimate the effect of treatment duration on survival outcomes using observational data. *BMJ.* 2018;360:k182.

2. Hernán MA, Brumback B, Robins JM. Marginal structural models to estimate the causal effect of zidovudine on the survival of HIV-positive men. *Epidemiology.* 2000;11(5):561-570.

3. Xiao Y, Abrahamowicz M, Moodie EE. Accuracy of conventional and marginal structural Cox model estimators: a simulation study. *Int J Biostat.* 2010;6(2):Article 13.

4. van der Laan MJ, Polley EC, Hubbard AE. Super learner. *Stat Appl Genet Mol Biol.* 2007;6:Article25.

5. Karim ME, Platt RW. Estimating inverse probability weights using super learner when weight-model specification is unknown in a marginal structural Cox model context. *Stat Med.* 2017;36(13):2032-2047.

6. Zhang Z. Multiple imputation with multivariate imputation by chained equation (MICE) package. *Ann Transl Med.* 2016;4(2):30.

7. White IR, Royston P, Wood AM. Multiple imputation using chained equations: Issues and guidance for practice. *Stat Med.* 2011;30(4):377-399.
